# Interpersonal communication, cultural norms, and community perceptions associated with care-seeking for fever among children under age five in Magoé district, Mozambique

**DOI:** 10.1186/s12936-023-04689-x

**Published:** 2023-09-21

**Authors:** Paul Hutchinson, Rose Zulliger, Jessica K. Butts, Balthazar Candrinho, Abu Saifodine, Thomas P. Eisele, Josh Yukich

**Affiliations:** 1 Department of International Health and Sustainable Development, Tulane School of Public Health and Tropical Medicine, New Orleans, LA USA; 2 U.S. President’s Malaria Initiative, U.S. Centers for Disease Control and Prevention, Maputo, Mozambique; 3 U.S. President’s Malaria Initiative, Malaria Branch, U.S. Centers for Disease Control and Prevention, Atlanta, USA; 4 National Malaria Control Programme, Ministry of Health, Maputo, Mozambique; 5 U.S. President’s Malaria Initiative, United States Agency for International Development, Maputo, Mozambique; 6 Center for Applied Malaria Research and Evaluation, Department of Tropical Medicine, Tulane School of Public Health and Tropical Medicine, New Orleans, LA USA

**Keywords:** Care-seeking, Malaria, Interpersonal communication, Norms, Attitudes

## Abstract

**Background:**

Malaria is endemic throughout Mozambique, contributing significantly to the country’s burden of disease. Prompt and effective treatment for fevers in children can limit the mortality and morbidity impacts of the disease but many children in the country are not taken for formal care when ill. Using an ideational model of behaviour, this study assesses the magnitude of the relationships for potential drivers of care-seeking, including interpersonal communication, malaria messaging, and knowledge and attitudes about malaria, with actual care-seeking behaviours for under-five children with fever in Magoé district, Mozambique.

**Methods:**

Data on the care-seeking behaviours for fever come from a 2019 household malaria survey in Magoé district. Households were randomly selected for interview from among those with at least one child under age five and one net for every two household members. From 1621 mother-child dyads, the analytical sample consists of 300 children under age five with a fever in the 2 weeks prior to the survey. Multilevel random effects logistic regression models are estimated to test for associations between care-seeking behaviours and hypothesized behavioural determinants, including interpersonal communication (IPC), malaria messaging, ideational factors (e.g., norms, attitudes, beliefs, risk perceptions), and community characteristics.

**Results:**

Overall, 18.5% of children under age five (N = 300) were reported to have fever in the previous 2 weeks and, of these, 68.5% were taken to a formal sector health care provider. Multivariate models highlight significant roles for interpersonal communication; care-seeking was highest among mothers who spoke only with friends/community members about malaria (94.0%, p < 0.001), followed by those who spoke only with their husband (78.6%, p = 0.015), relative to 63.3% who spoke with no one. Care-seeking decisions made by a child’s grandmother were associated with a 25.0% point (p = 0.001) greater likelihood of seeking care relative to decisions made by the mother alone. Exposure to any malaria messaging was also positively associated with care-seeking (90.5% versus 62.7%, p < 0.001). In contrast, among all individual- and community-level ideational factors, only perceptions of self-efficacy to seek care were related to care-seeking behaviours.

**Conclusions:**

These results suggest that social and behaviour change interventions that focus on encouraging families and community members to talk about malaria and the need to promptly seek treatment for fevers in children may be particularly effective at increasing this behaviour in this and similar settings. Such messaging and IPC should consider grandmothers as a target audience, as they appear to be perceived as highly influential in care-seeking decision-making in this community.

**Supplementary Information:**

The online version contains supplementary material available at 10.1186/s12936-023-04689-x.

## Background

Malaria is endemic throughout Mozambique. In 2020, over 11 million malaria cases were diagnosed by public health services [1]. Currently, malaria accounts for 29% of all deaths and 42% of deaths in children under the age of five [2]. Anti-malarial artemisinin-based combination therapy (ACT) is highly effective in preventing malaria deaths [3], yet adequate malaria care-seeking behaviours are far from universal in Mozambique. According to the 2018 Malaria Indicator Survey [4], only 69% of children under 5 years of age with a fever in the past two weeks sought treatment and even fewer, 48%, had blood taken for malaria testing. Once a child with fever is taken for care in the formal health sector, a point of care test for malaria is national policy, followed by treatment with ACT if positive. Children who do not receive such prompt and effective diagnosis and treatment are at greater risk of experiencing severe malaria and consequently at greater risk of dying. It is, therefore, critical to gain a better understanding of why children with a fever are, or are not, taken for care.

This study focuses on Magoé district, located in the southwest region of Tete province in Mozambique, an area considered to have a high burden of malaria [5]. The district reported 26,604, 28,889 and 10,077 malaria cases in 2019, 2020 and 2021, respectively, among an estimated population of 98,000 people (INE, 2021). Coverage of community radio is high, and in 2020 the district benefited from an ITN campaign. However, the district did not receive indoor residual spraying (IRS) in the 3 years immediately prior to the study.

**Fig. 1:**
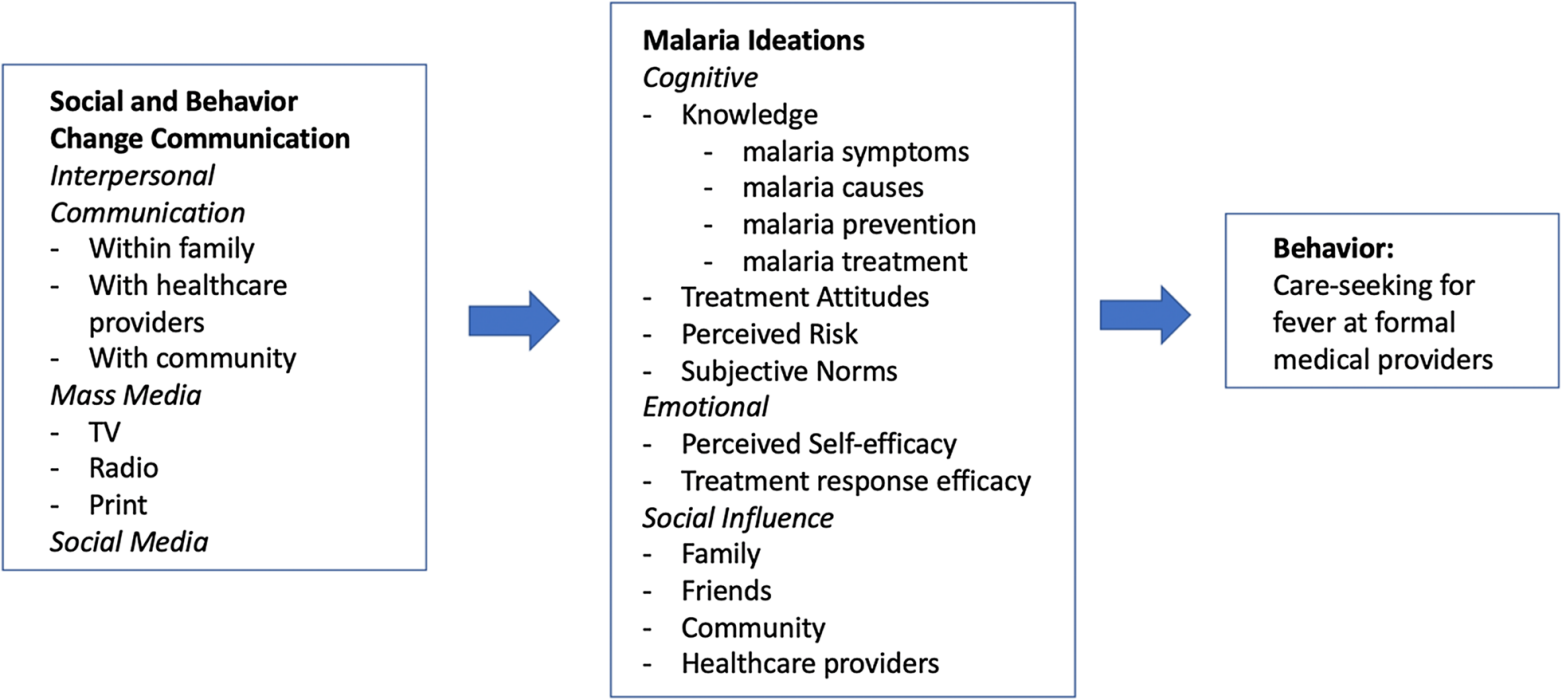
Ideational model of strategic communication and behavior change

In order to identify important influencers or impediments to prompt and effective treatment of child fevers, this work applies the Ideational Model of Strategic Communication and Behaviour Change [6–10] to identify and estimate the strength of associations between ideational “factors” and care-seeking at a formal healthcare provider by mothers of children under age five with fever. The Ideational Model merges components of multiple behavioural theories, including the Diffusion of Innovations [11], the Theory of Planned Behavior [12], Social Cognitive Theory [13, 14], and the Transtheoretical Model [15]. In this framework (Fig. 1), people’s decisions about care-seeking for fever are hypothesized to be influenced indirectly by malaria social and behaviour change communication (SBCC) (e.g., mass media, social media) and interpersonal communication (IPC) among important social influences (e.g., family, friends, community members and healthcare providers). These catalysts work to shift precursors to behaviour change, such as malaria knowledge, attitudes, treatment-seeking norms, and self-efficacy to engage in care-seeking care for fever. SBCC and IPC are intended to improve knowledge and awareness of malaria symptoms, appropriate treatment, and prevention mechanisms, as well influence perceptions about the potential risks of malaria and its severity and susceptibility. Mass media (e.g., advertisements, dramas, printed materials) is used to model or demonstrate appropriate treatment behaviours, while personal experiences of key social influencers can shift norms, attitudes, beliefs related to impediments to care-seeking, such as the concerns about the quality of providers, the efficacy of drugs, or the need for diagnostic tests. At the same time, social influences may be detrimental to care-seeking behaviours, propagating or perpetuating myths and inaccurate information about malaria transmission, prevention, and treatment.

Empirical applications of the Ideational Model to trace and test the pathways from SBCC exposure and interpersonal communication to changes in ideational factors and ultimately to care-seeking behaviour for malaria are limited. In one application of the model, Do and colleagues [10] identify treatment-seeking social norms – perceptions of what others in the community do when a child is sick with fever—and malaria knowledge as key factors driving decisions to seek care for a child’s fever. Other factors, such as risk perceptions and attitudes towards healthcare providers, were not found to be associated with care-seeking. A study in Guyana [16] assessed the influence of ideational factors (e.g., general malaria knowledge, perceived severity, perceived susceptibility, beliefs, perceived self-efficacy, perceived norms, interpersonal communication, and perceived response efficacy) on the malaria care-seeking behaviours of miners. The authors identified a positive association between an additive index of ideational factors and the likelihood of seeking care, but the use of an aggregated measure prevented assessments of the relative strength of associations between specific ideational factors and care-seeking.

Researchers have also applied the Ideational Model to other malaria behaviours, such as the uptake of intermittent preventive treatment in pregnancy (IPTp) and the use of insecticide-treated bed nets. Often these analyses have been limited in their conclusions. Awantang and colleagues [17] identified greater malaria knowledge and more favourable norms as factors associated with IPTp uptake but did not find evidence of an effect from more accurate malaria risk perceptions or greater self-efficacy. In a study of bed net usage in Nigeria, Okoh et al., [18] focused on the aggregated sub-components of the Ideational Model—cognitive, emotional and social domains, as shown in Fig. 1—and found that these constructs have a “significant positive effect on consistent bed net use,” implying a causal relationship even though the use of cross-sectional data prevents a rigorous examination of time-order of effects. Specific components of each of these sub-domains were not analysed. Similarly, Storey et al. [19] employed an Ideational Model of bed net use in Madagascar, Mali and Nigeria to estimate the associations between fourteen different ideational factors and net use, as well as between an additive index of the fourteen ideational factors and net use. The authors noted that higher ideational index scores yielded greater likelihoods of net use but found, among the fourteen ideational factors, only “perceived self-efficacy to purchase enough nets” to be positively and statistically significantly related to net use across all countries.

Social influences, particularly husbands have also been linked with the likelihood of care-seeking in a variety of contexts and for a variety of health behaviours [20, 21], but little is known about how IPC between spouses and within larger social networks impact care-seeking. Do and colleagues were unable to find evidence that general malaria discussions influence care-seeking. However, their models tested only for the effects of discussions with anyone and did not distinguish between conversations with different potential participants (e.g., husbands, family members, or others).

Another factor that may influence care-seeking behaviours is the autonomy of women, who are generally the principal caregivers for children. Studies have found that women are frequently excluded from decision-making about healthcare [22], including for malaria [23, 24], and often constrained by unbalanced gender roles and patriarchal decision-making structures [25]. This can further limit care-seeking behaviours as greater autonomy in decision-making, often measured as participation in common household decisions, and greater intra-household bargaining power, have consistently been shown to be positively associated with health behaviours [26–29].

The extant literature therefore identifies numerous factors present in the Ideational Framework that have been shown to influence the decision to seek care for childhood illnesses and other health ailments. However, the evidence base surrounding individual-level ideational components has often been mixed, with some factors being of great import in certain contexts but not in others. This study, therefore, aims to expand the evidence base surrounding the behavioural drivers of care-seeking for fever among a population in a highly malaria-endemic area of Mozambique. The focus is on estimating the magnitude of the relationships between malaria SBCC, ideational factors, IPC, and community-level influences on care-seeking in order to better guide the design of SBCC programmes.

This study has several objectives:

1. To identify factors from the Ideational Model that are associated with care-seeking behaviours for under-five children with fever, focusing on: (1) individual-level ideational factors (e.g., norms, risk perceptions, knowledge, attitudes); (2) malaria IPC within households and social networks; (3) women’s decision-making autonomy; and (4) exposure to different types and sources of SBCC interventions (e.g., mass media, print media, CHWs, implementing partners).
2. To test whether women’s decision-making autonomy modifies the effects of IPC on appropriate care-seeking behaviours for children’s fevers;
3. To determine if community-level ideational factors (e.g., norms and perceptions held by the community) have independent associations with care-seeking behaviours once individual- and household-level ideational factors are controlled for.

## Methods

### Study site

The study was conducted in Magoé District, Mozambique. The study was originally intended as an unblinded, three-arm, parallel-groups, cluster randomized controlled trial (CRCT) to test the effectiveness of mass media and on-the-ground health communication activities (e.g., community dialogues, health talks, influencer meetings) to improve the level and consistency of bed-net use. Magoé District was chosen for the study because it has a high burden of malaria and is located in Tete province, one of the targeted provinces for implementation of the Integrated Malaria Programme (IMaP), Mozambique’s flagship partner of the President’s Malaria Initiative (PMI).

That trial was interrupted because of COVID-19 after the baseline data used in this analysis were collected. As part of the data collection, a set of census enumeration areas were selected for use as study clusters for randomization using a bespoke genetic algorithm designed to identify study clusters with sufficient households for trial inclusion and having minimal contact with other selected clusters in order to minimize contamination in the trial.

### Enumeration and sampling

Prior to the selection of households for participation in the household survey, a full enumeration of all households in selected study clusters was conducted. The purpose of the enumeration was to identify households meeting eligibility criteria for inclusion in the study. Households were eligible for participation in the study if they had at least one child under 5 years of age and at least one LLIN for every two children under 5 years of age. In each enumeration area, 15 households meeting study inclusion criteria were selected using simple random sampling for participation in the household survey. In each household meeting the eligibility criteria, interviews were conducted with the household head and all women of reproductive age (15–49 years).

### Sample size calculations

The sample size for this study was driven by the expected power to detect differences across the three study arms at end-line for the following two outcomes:

1. The proportion of de facto resident children under 5 years of age that slept under any net the previous night, among those in households with at least 1 net for every 2 de facto household residents under five; and.
2. The proportion of de facto resident persons that slept under any net the previous night, among those in households with at least 1 net for every 2 de facto household residents.

Sample size calculations were based on data from the 2015 Mozambique AIDS Indicator Survey, restricted to data only from Tete province. For Tete province for the two primary outcomes, intracluster correlation coefficient (ICC) estimates ranged from 0.126 for under five children to 0.165 for all ages. The estimates of usage of nets among households with at least one net per every two under five children in these groups were 61% for under five children and 63% for all ages. For the purpose of calculations, a value of 0.15 was used for the ICC, and baseline/control group net use prevalence was assumed to be 70%. All calculations assumed 80% power and 5% alpha level in a two-sided test. As such, a 40 cluster per arm trial was intended to have 80% power to detect an increase from 70% to ~ 82% use of nets. To achieve the required fifteen observations of under five children in households with at least one net for every two *de facto* under five child residents in each cluster, it was necessary to screen a larger number of households to be sure that they met the inclusion criteria during enumeration.

### Survey design and content

Data were collected using enumerator-directed face-to-face interviews with household heads and mothers of children with a child under 5 years. Two questionnaires were used for the data collection: a household questionnaire and a questionnaire for women of reproductive age (WRA: 15–49 years). The household questionnaire was modelled after a standard Malaria Indicator Survey questionnaire [30].

The woman’s questionnaire was modelled after the Malaria Behaviour Survey and collected information on the background characteristics of women of reproductive age, birth histories, children’s net use, pregnancy and intermittent preventive treatment of malaria in pregnancy, autonomy and decision-making efficacy, access to media, exposure to malaria SBCC, and IPC about malaria [31]. Questions were also asked about components of the Ideational Model, including knowledge about malaria, prevention of malaria during pregnancy, mosquito net use, and care-seeking; perceived susceptibility to malaria, norms related to care-seeking, attitudes, response efficacy, and self-efficacy.

### Data

A total of 1800 households were selected for the survey, and data were collected from 1722 women of reproductive age (15–49 years) on malaria-related knowledge, attitudes, behaviours, and outcomes, including for their children under the age of 5 years (e.g., LLIN use, fever, treatment). A total of 1621 mother-child dyads were identified in the data. Among these 1621 dyads, there were 300 children (18.5%) with fever in the two weeks preceding the survey, thereby representing the effective sample for this study.

### Data analysis

Univariate analysis of means and proportions are reported after accounting for the cluster survey design utilizing the Huber-White Sandwich estimator for ascertaining empirically estimated standard errors. Multivariable analysis was conducted using multi-level logistic regression models including random effects for study cluster.

The primary outcome for the analysis was care-seeking, categorized as binary variable for whether a child was taken to a formal sector healthcare provider for treatment of fever. Formal health care providers included hospitals, health units, health centres, mobile clinics, CHWs, private/clinic hospitals, and private doctors. Nearly all formal sector providers are public as the private medical sector in Mozambique is quite small. Regression models controlled for basic socio-demographic characteristics of the child, the mother and the household, including the age of the child (in complete years), the sex of the child, the age of the mother (15–19, 20–29, 30–39, and 40–55), the mother’s highest grade completed (none, some primary, completed primary, some secondary, secondary or more), and wealth. Wealth was measured using an asset-based measure constructed from ownership of key consumer durables and then compiled into an index using principal components analysis (PCA) [32]. Households were categorized into quintiles from poorest to wealthiest.

The explanatory variables for assessing the primary research questions come from the extensive battery of questions in the women’s questionnaire on behavioural determinants, exposure to media and malaria messaging, and IPC with others about malaria. Variables in the model are intended to capture the essential components of the Ideational Model, including malaria IPC, knowledge, self-efficacy, decision-making autonomy, risk perceptions, descriptive norms, attitudes, and exposure to SBCC.

IPC about malaria was measured from two variables: “In the last 6 months, did you talk about malaria with your spouse or partner?” and “In the last 6 months, did you talk about malaria with your friends or community members?”

Several survey questions addressed general malaria knowledge, including “*What are the signs and symptoms of malaria?*”, “*How do you get malaria*?”, “*What can one do to avoid malaria?*”, “*Does malaria have a cure?*” and “*What medicines can be used to cure malaria?*” An index created using principal components analysis (PCA) had a low Cronbach’s alpha and was not utilized. Instead, to avoid issues of multicollinearity, one knowledge question was included in the regression modelling: a binary variable indicating if a mother knows that a mosquito net can prevent malaria transmission.

Measures of self-efficacy to get permission to take a child for care and care-seeking autonomy were constructed from two questions, “*Can you get permission from your husband or other family member to take your child to the health facility/health worker when you think your child has malaria*?” and “*In your household, who usually makes decisions about what to do when your children are sick?*” (Respondent, spouse, joint decision with spouse, mother, mother-in-law, someone else).

For risk perceptions, descriptive norms, attitudes and response efficacy, scales were developed to transform Likert-scale questions using a five-point agreement scale (strongly agree to strongly disagree, including don’t know) or frequency scale (always to rarely, including don’t know) into constructs in the Ideational Model. Ideational scales were synthesized into unidimensional quantitative scores using the *polychoricpca* PCA command [33]. Prior to scale construction, scale cohesion of the individual components was assessed using Cronbach’s alpha.

General malaria risk perceptions were derived from eight questions such as “*Nearly every year, someone in this community gets a serious case of malaria*.” General malaria norms were derived from four questions including “*most people in this community talk about malaria with their families*.” A measure of care-seeking attitudes was constructed from fifteen Likert-scale questions, including “*the health provider is always the best person to talk to when you think your child may have malaria*.” Perceived response efficacy was estimated from four questions, such as “a *person should still take malaria medicine even if the malaria test result says that the fever is not due to malaria*.” Finally, an index of care-seeking norms was constructed from questions such as “*in your community, how frequently do health facilities have the tests for malaria*?” For each of these constructs, women were categorized into those above the mean for the scale (“High”) and those below the mean (“Low”).

To gauge exposure to malaria SBCC, a question was asked about whether a woman had seen or heard any messages about malaria in the previous 6 months and, if so, the source of the messages. Sources were categorized into formal medical (e.g., government), other medical (e.g., pharmacy), community-based (e.g., community dialogue), home visit, mass media, and phone/SMS.

Community-level variables were constructed from the individual-level ideational scales. Specifically, means within communities for all individual-level constructs were calculated (e.g., mean level of malaria risk perceptions). Communities were then divided into those with higher than mean values of individual constructs and lower than mean values.

To assess the potential effects of multicollinearity, in post-estimation analysis the uncentered variance inflation factors for the explanatory variables contained in the regression model were calculated. This provided a measure of the extent to which each explanatory variable was affected by other correlated variables in the model. In no cases did the vif exceed 3, indicating levels of collinearity that were unlikely to be problematic.

## Results

Among the 300 children under five with a fever in the previous 2 weeks, 60.5% were male. Approximately 60% of mothers had less than a primary education (Table 1). The majority of women whose children had a recent fever, 63.9%, did not report talking about malaria either with her husband or with friends and community members in the last 6 months, while 18.4% had talked with her husband only, 8% had talked with friends/community members only, and 9.7% had spoken with both. Only 21.4% of women reported being the sole decision-makers about seeking care for sick children. In 35% of cases, the father usually made such decisions, while 36% of couples made the decision jointly. Most women, 88.6%, reported they could get permission from a husband or other family member to seek care for a sick child.

**Table 1:** Characteristics of households and household respondents from the 2019 baseline survey, Magoé district, Mozambique

	%Freq.	n	
Total n		299	
Child’s age (years)			
0	18.7	56	
1	22.1	66	
2	16.7	50	
3	18.1	54	
4	24.4	73	
Child’s sex			
Female	39.5	118	
Male	60.5	181	
Mother’s Age (years)			
15–19	17.1	51	
20–29	48.8	146	
30–39	26.1	78	
40–55	8.0	24	
Mother’s Level of Education			
None	19.1	57	
Some Primary	41.1	123	
Completed Primary	19.7	59	
Some Secondary	15.4	46	
Secondary or more	4.7	14	
Wealth Quintile			
Poorest	19.4	58	
2nd Poorest	13.7	41	
Middle	21.7	65	
2nd Wealthiest	21.1	63	
Wealthiest	24.1	72	
Talked with someone about malaria in last 6 months			
No one	63.9	191	
Husband only	18.4	55	
Friends only	8.0	24	
Husband and Friends	9.7	29	
In your household, who usually makes decisions about what to when your child is sick with fever?			
Mother/respondent	21.4	64	
Father	35.1	105	
Joint	36.5	109	
Grandmother	5.4	16	
Other	1.7	5	
Can you get permission from your husband or other family member to take your child for care?			
DK/could not	11.4	34	
Could	88.6	265	
Knows signs or symptoms of malaria: FEVER			
Yes	65.9	197	
Knows malaria transmitted by mosquito bites			
Yes	90.0	269	
Knows that nets can protect against malaria			
Yes	90.3	270	
Knows that medicines can be used to cure malaria: ARTEMISININ COMBINATION THERAPY (ACT/COARTEM)			
Yes	75.5	206	
Seen or heard any messages about malaria in past 6 months?			
Yes	23.1	69	
Source			
SMS	7.0	21	
Government health facility	18.4	55	
Other medical	0.7	2	
Comm. leader mtg, savings club, mosque/church	1.0	3	
Home visit	2.7	8	
TV, radio, posters, newspaper	4.0	12	

Malaria knowledge was high amongst mothers. Approximately 90% of mothers knew that malaria is transmitted by mosquito bites and that nets can protect against malaria. Two-thirds of women identified fever as a symptom of malaria, and three-quarters knew that ACT (or the brand name Coartem for artemether-lumefantrine) can cure a case of malaria.

Nearly a quarter of women had seen or heard a malaria message in the past six months, most commonly from a medical source such as a health facility, a mobile clinic, or a community health worker (CHWs) (18.4%). Other sources included SMS text messages (7.0%), home visits (2.7%), or through mass media (4.0%).

Of the 300 under-fives in the sample with recent fever, 205 (68.5%) were taken for care at a formal health care provider. This is nearly identical to the percentage (69%) of children with fever in the past two weeks taken for care in the 2018 MIS [4]. In bivariate analyses, several ideational factors were associated with care-seeking for fever, Additional details on the characteristics of households and household respondents are in Table S1.

The multivariable regression model for care-seeking (Table 2) highlights significant associations with interpersonal communication and self-efficacy to seek care, as well as exposure to malaria SBCC, but not for many of the other ideational factors. For example, IPC with spouses and IPC with friends/community members were positively associated with care-seeking behaviours relative to not speaking with anyone. However, there was no evidence that the combination of the two, IPC with both spouses and friends/community members, increased the likelihood of seeking care relative to speaking with no one.

**Table 2:** Adjusted odds ratios from random-effects logistic regression models for care-seeking examining IPC autonomy, malaria knowledge, general and specific malaria messaging, malaria ideations and community characteristics, from the 2019 baseline survey, Magoé district, Mozambique

Variable	Odds ratio	95% conf.	Interval	P > z
Age of child				
0 years	1.000			
1 year	0.586	0.167	2.060	0.404
2 years	0.730	0.184	2.896	0.654
3 years	1.870	0.484	7.227	0.364
4 years	0.410	0.118	1.416	0.159
Sex of child				
Female	1.000			
Male	0.797	0.347	1.831	0.593
Age of Mother				
15–19 years	1.000			
20–29 years	1.152	0.349	3.807	0.816
30–39 years	1.767	0.481	6.495	0.392
40–55 years	2.079	0.361	11.958	0.412
Mother’s education				
None	1.000			
Some primary	1.851	0.584	5.864	0.295
Completed primary	1.851	0.424	8.084	0.413
Some Secondary	0.686	0.133	3.532	0.652
Secondary or more	0.886	0.087	9.024	0.919
Wealth quintile				
Poorest	1.000			
2nd poorest	4.244	0.815	22.096	0.086
Middle	4.585	1.122	18.734	0.034
2nd wealthiest	2.718	0.681	10.849	0.157
Wealthiest	7.588	1.440	39.996	0.017
IPC about malaria				
Spoke with no one	1.000			
Spoke only with husband	3.688	0.973	13.982	0.055
Spoke only with friends/community	25.591	1.704	384.297	0.019
Spoke with both	3.471	0.698	17.270	0.128
Usual decider on care-seeking				
Respondent	1.000			
Spouse	0.923	0.288	2.959	0.893
Joint	1.067	0.337	3.384	0.912
Grandmother	15.364	1.459	161.770	0.023
Other	1.278	0.033	48.773	0.895
Self-efficacy to seek permission				
No/don’t know	1.000			
Yes	4.117	1.096	15.459	0.036
Knows nets as prevention				
No	1.000			
Yes	3.339	0.792	14.077	0.101
Exposure to malaria messaging				
No				
Yes	19.018	3.915	92.382	0.000
Malaria Ideations				
General malaria risk perceptions				
Low	1.000			
High	1.357	0.570	3.226	0.490
General malaria norms				
Low	1.000			
High	0.712	0.300	1.691	0.441
Treatment attitudes				
Low	1.000			
High	0.950	0.378	2.387	0.912
Treatment response efficacy				
Low	1.000			
High	1.618	0.604	4.339	0.339
Treatment seeking norms				
Low	1.000			
High	1.303	0.548	3.098	0.549
Community Variables				
High exposure community	0.618	0.212	1.801	0.377
Positive treatment attitudes	1.119	0.370	3.389	0.842
Positive response efficacy	0.546	0.191	1.558	0.258
High self-efficacy	1.914	0.642	5.701	0.244
High treatment seeking norms	2.579	0.804	8.265	0.111
High malaria susceptibility	0.550	0.186	1.629	0.280
High malaria severity	1.701	0.580	4.985	0.333
Constant	0.010	0.000	0.782	0.038
Var(Constant)	1.978	0.518	7.557	
LR test v logistic model				
Chibar2(01)	7.06			
Prob ≥ chibar2	0.004			
Observations	299			
Number of groups	100			

Self-efficacy to seek permission for care, as measured by the ability to get permission to take a child suspected of having malaria to a health facility, was positively associated with care-seeking. In contrast, there were no significant differences in care-seeking behaviours for children whose parents made joint decisions or for children whose fathers generally made such decisions, compared to cases where the mother alone made the decision. However, when grandmothers were the principal decision-makers, children were more likely to be taken for care. Additional models were run to test whether the effects of communication were modified by greater autonomy, interacting the IPC variable with both the decision-making variable and the self-efficacy variable. None of these interactions were statistically significant, indicating that there is not a combinatory effect of IPC and autonomy. Therefore, those results are not presented here.

The study found no evidence of significant associations between care-seeking and the remaining ideational factors. Specifically, the variable measuring knowledge of nets as a malaria prevention mechanism was not significantly related to care-seeking, possibly due to the high levels of malaria knowledge and minimal variance across respondents. Tests with variance inflation factors indicated the absence of significant multicollinearity among the knowledge variables, suggesting that the regression coefficients related to these variables are less likely to be attenuated. Additionally, the study found that malaria risk perceptions, malaria norms, treatment attitudes, treatment response efficacy, and care-seeking norms did not show statistically significant relationships with care-seeking.

Seeing or hearing any messages about malaria in the prior 6 months, regardless of source, was positively associated with care-seeking. To further identify the relative effectiveness of different sources of information, we ran a separate model distinguishing among five sources of exposure: SMS message, health facility, other medical sources, community source, home (visits), and from mass media. Several of these variables were perfectly correlated with care-seeking and hence their odds ratios could not be calculated.

None of the community-level variables were significantly related to care-seeking.

To provide a better sense of the importance of the ideational and communication variables, Table 3 re-interprets the odds ratios from the regression model as adjusted probabilities of seeking care and average marginal effects, omitting the results for the community variables for parsimony. As noted above, relative to speaking with no one, talking with a husband and talking with friends/community members about malaria in the prior 6 months were both positively related with care-seeking, associated with increases of 16.3% points (p = 0.015) and 31.7% points (p < 0.001), respectively, relative to speaking with no one. Speaking with both a husband and friends/community members showed no additional effect relative to speaking with husbands alone.  Care-seeking decisions made by a child’s grandmother were associated with a 25.0% point (p = 0.001) greater likelihood of seeking care compared to when the mother decided alone, although only 5% of such decisions (N=16) were made solely by grandmothers, as per Table 1. Greater self-efficacy to obtain permission to seek care was associated with an 18.3% point difference in care-seeking (70.5% versus 52.2%, p = 0.026). The effects of all other malaria ideations (e.g., risk perceptions, treatment attitudes, norms, and response efficacy) were not statistically associated with care seeking. Having heard messages about malaria was associated with a 27.8% point greater likelihood of care seeking – 90.5% versus 62.7% (p < 0.001). However, among those who reported exposure to malaria messaging, 83% of the sample were exposed at a government health facility, perhaps while seeking treatment.

**Table 3:** Adjusted probabilities and average marginal effects assess IPC autonomy, malaria knowledge, general and specific malaria messaging, malaria ideations and community characteristics, from the 2019 baseline survey, Magoé district, Mozambique

Variable	Adjusted probability	Average marginal effect (% point)	P > z	95% conf. (%)	Interval
Age of Child					
0 years	72.3%				
1 year	64.2%	− 8.1	0.267	− 22.4	6.2
2 years	68.2%	− 4.2	0.606	− 20.0	11.7
3 years	79.3%	7.0	0.318	− 6.7	20.8
4 years	61.7%	− 10.6	0.144	− 24.8	3.6
Sex of Child					
Female	70.5%				
Male	67.2%	− 3.2	0.512	− 12.9	6.4
Age of Mother					
15–19 years	65.4%				
20–29 years	67.1%	1.7	0.811	− 12.3	15.7
30–39 years	71.0%	5.7	0.465	− 9.5	20.8
40–55 years	73.3%	7.9	0.428	− 11.6	27.4
Mother’s Education					
None	64.7%				
Some primary	71.3%	6.6	0.352	− 7.3	20.4
Completed primary	72.4%	7.7	0.381	− 9.5	24.9
Some Secondary	59.1%	− 5.7	0.564	− 24.9	13.6
Secondary or more	60.2%	− 4.5	0.757	− 33.0	24.0
Wealth Quintile					
Poorest	52.7%				
2nd poorest	72.5%	19.8	0.035	1.4	38.3
Middle	71.8%	19.0	0.022	2.7	35.4
2nd wealthiest	68.0%	15.3	0.079	− 1.8	32.4
Wealthiest	78.0%	25.2	0.005	7.6	42.9
IPC about malaria					
Spoke with no one	62.3%				
Spoke only with husband	78.6%	16.3	0.015	3.2	29.4
Spoke only with family members/ friends/community	94.0%	31.7	0.000	19.0	44.4
Spoke with both	77.1%	14.8	0.108	− 3.3	32.9
Usual decider on care-seeking					
Respondent	67.0%				
Spouse	66.0	− 1.0	0.891	− 14.8	12.9
Joint	68.0%	1.0	0.882	− 12.5	14.5
Grandmother	92.0%	25.0	0.001	10.3	39.7
Other	67.0%	0.0	1	− 44.6	44.6
Self-efficacy to seek permission					
No/Don’t Know	52.2%				
Yes	70.5%	18.3	0.026	2.1	34.4
Knows nets as prevention					
No	54.3%				
Yes	70.0%	15.6	0.087	− 2.3	33.5
Exposure to malaria messaging					
No	62.7%				
Yes	90.5%	27.8	0.000	18.1	37.6
Malaria Ideations					
General malaria risk perceptions					
Low	66.5%				
High	70.2%	3.8	0.468	− 6.4	13.9
General malaria norms					
Low	71.3%				
High	66.6%	− 4.7	0.347	− 14.5	5.1
Treatment attitudes					
Low	68.9%				
High	67.9%	− 1.0	0.852	− 11.6	9.6
Treatment response efficacy					
Low	66.8%				
High	72.7%	5.9	0.298	− 5.2	17.1
Treatment seeking norms					
Low	66.2%				
High	70.7%	4.5	0.398	− 5.9	14.8

## Discussion

This study found that close to 70% of children under five with fever are taken for care and that many factors in the Ideational Model are associated with that decision. Testing the relationships with care-seeking for factors in the emotional domain of the Ideational Model, it was found that women in this area of Mozambique have reasonably high self-efficacy to obtain permission to take their child for treatment when the child has a fever, and those with reported higher self-efficacy, are more likely to do so. This finding is supported by previous studies on ideational factors associated with care-seeking for fevers [34], as well as more broadly from a systematic review on care-seeking across countries in Africa that found self-efficacy to be a key factor for care-seeking for fever, diarrhoea, and suspected pneumonia in children [35]. It is also notable that beyond a mother’s own perceived ability to decide about seeking treatment for her child’s fever, the child’s grandmother appeared to be highly influential on whether treatment was sought for a child’s fever, although the precise pathway of influence remains unclear. This finding has been noted in qualitative research from other high burden settings in Mozambique, indicating that grandmothers may be an important target population for SBC interventions [36].

No statistically significant relationships were identified between cognitive factors in the Ideational Model (e.g., knowledge, attitudes, risk perceptions, and subjective norms) and care-seeking for fever. While this result may indicate that these factors are not as important as other factors in determining care-seeking, it may also reflect possible sample selection bias. As noted previously, participation in the study was determined by ownership of the WHO-recommended one LLIN for every two under-five children, a household decision that may be associated with many of the same ideational factors as those for care-seeking. Relative to the population at large, this sample could plausibly be more likely to exhibit other positive malaria preventive and treatment practices and to possess greater malaria knowledge, more positive attitudes towards malaria prevention and treatment, and greater acceptance of norms of care-seeking for fevers. Lesser variation in these factors as a result of the selection process may bias regression estimates towards the null hypothesis of no effect.

Social factors in the Ideational Model, as measured by whether a woman spoke about malaria with a spouse or with friends/community members, were positively associated with the likelihood of taking a sick child for care. However, there was no evidence of additive effects of speaking to both, potentially indicating that any form of IPC about malaria is beneficial. The positive effect of IPC with spouses may also be reflected in the variable on self-efficacy to obtain permission to seek care, given that only one in five women are the sole decision-makers about care-seeking. Along with the finding that exposure to SBCC messaging on malaria was positively associated with care-seeking, the finding that engaging in IPC with community members is influential in care-seeking decisions suggests that targeted IPC interventions may be highly effective at increasing the behaviour to seek care for a child’s fever. This is supported by a study by Do and colleagues [10] that showed that perceptions of what others in the community do when a child is sick with fever is a key factor driving decisions to seek care. Beyond care-seeking specifically for a fever, these findings are further supported by research that has shown that social influences, particularly the role of husbands and the level of a woman’s decision-making autonomy, have been linked with the likelihood of care-seeking in a variety of contexts and for a variety of health behaviours [20, 21].

There are several notable limitations of this study. First, because it is difficult to ascertain temporality with cross-sectional data, the factors that were found to be associated with a mother’s care-seeking for her child’s fever cannot be determined to be causal, and more specifically reverse causality may be present. For example, the finding that perceived self-efficacy is associated with care-seeking may indicate that mothers who sought care for their children may have subsequently felt more self-efficacious for doing so and responded as such during the survey interview. Hence, because the data are retrospective, it is possible that feelings of self-efficacy were the result of seeking care rather than a determinant of seeking care.

Second, women were asked to self-report about whether they took their child for treatment for their fever, as well as where they sought care. While such information ascertained from household surveys has been shown to be accurate, there is always the threat of recall error and bias [37–39].

Third, mother’s reporting on self-efficacy, autonomy, and malaria-related ideations related to her ability to seek care for her child’s fever may have been subject to social desirability bias where she presented these factors in a more favourable light. Finally, the household survey in this study was conducted in November which is at the end of the dry season in Mozambique when malaria is at its lowest, which may have impacted the level of care-seeking as well as mothers’ perceptions about care-seeking for her child with a fever, as compared to the rainy season when malaria is more prevalent.

Finally, the population from which data were collected, which was tied to having the recommended one LLIN for every two children under the age of 5 years, may represent a very select set of households with better malaria knowledge, including greater awareness of malaria risks, severity, and susceptibility. Reduced variation in these factors could attenuate our regression model coefficients towards the null of no effects. Hence, it is possible that some of these results could actually underestimate the true effects of these factors relative to those for the general population.

## Conclusion

Access to early diagnosis and treatment of uncomplicated malaria is a cornerstone of case management and a key intervention for preventing severe malaria and death, especially in children [40]. However, in many malaria-endemic countries, prompt care-seeking remains low. Understanding which factors promote care-seeking is critical to improve malaria case management because access to early diagnosis and treatment of uncomplicated malaria are cornerstones of controlling and eventually eliminating malaria.

This study’s examination of care-seeking behaviours for children under five with fever in Magoé district, Mozambique has led to several important conclusions. First, general messaging on malaria was shown to increase care-seeking for childhood fevers in this setting. Moreover, a mother’s interaction with her spouse and community were also influential in her decision to seek treatment for the fever. Taken together, SBCC interventions that focus on getting families and their community to talk about malaria and seeking prompt treatment for suspected malaria and fevers may be particularly effective in similar settings. Second, such messaging and IPC may be particularly effective if grandmothers are involved, as they appear to be perceived as highly influential in care-seeking decision-making in this community. Lastly, SBCC, especially IPC, that attempts to bolster a mother’s perception that she is in control of treatment seeking may be influential in improving her treatment-seeking for her child’s fever.

### Supplementary Information

**Additional file 1 : Table S1.** Characteristics of households and household respondents from the 2019 baseline survey, Magoé district, Mozambique (N = 299).

## Data Availability

The datasets used and/or analysed during the current study are available from the corresponding author on reasonable request.
