# Supplementary material for: Interpersonal communication, cultural norms, and community perceptions associated with care-seeking for fever among children under age five in Magoé district, Mozambique
Source: Malar J. 2023 Sep 21;22:279. doi: 10.1186/s12936-023-04689-x (PMC10515048; doi:10.1186/s12936-023-04689-x)
Supplement: Supplementary file 1 — Additional file 1 : Table S1. Characteristics of households and household respondents from the 2019 baseline survey, Magoé district, Mozambique (N = 299). [file 12936_2023_4689_MOESM1_ESM.docx]

**Supplemental information**

SI Table 1. Additional characteristics of households and household respondents from the 2019 baseline survey, Magoé district, Mozambique (N = 299)

|  | **%** | **n** |  |
| --- | --- | --- | --- |
| Received malaria SMS messages in last 6 months | 7.02 | 21 |  |
| Saw malaria messages at government health facility/hospital, brigada movel, agentes polivalentes elementares | 18.39 | 55 |  |
| Saw malaria messages at pharmacy, informal market, traditional medicine/healer polivalentes elementares | 0.67 | 2 |  |
| Heard malaria messages at community-leader meeting, savings club meeting, mosque/church, community dialogue session | 1.00 | 3 |  |
| Heard malaria messages at home | 2.68 | 8 |  |
| Saw/heard malaria messages on mass media (posters, television, radio, newspaper) | 4.01 | 12 |  |
| **Nearly every year, someone in this community gets a serious case of malaria** | | | |
| Strongly agree | 85.62 | 256 |  |
| Somewhat agree | 6.69 | 20 |  |
| Somewhat disagree | 2.01 | 6 |  |
| Strongly disagree | 2.34 | 7 |  |
| Don't Know | 3.34 | 10 |  |
| **When your child has a fever, you almost always worry that it might be malaria** | | | |
| Strongly agree | 55.18 | 165 |  |
| Somewhat agree | 23.41 | 70 |  |
| Somewhat disagree | 9.36 | 28 |  |
| Strongly disagree | 9.70 | 29 |  |
| Don't Know | 2.34 | 7 |  |
| **During the rainy season, you worry almost every day that someone in your family will get malaria** | | | |
| Strongly agree | 77.26 | 231 |  |
| Somewhat agree | 13.38 | 40 |  |
| Somewhat disagree | 5.35 | 16 |  |
| Strongly disagree | 2.68 | 8 |  |
| Don't Know | 1.34 | 4 |  |
| **During the dry season, you worry almost every day that someone in your family will get malaria** | | | |
| Strongly agree | 52.51 | 157 |  |
| Somewhat agree | 24.08 | 72 |  |
| Somewhat disagree | 14.38 | 43 |  |
| Strongly disagree | 8.03 | 24 |  |
| Don't Know | 1.00 | 3 |  |
| **Young children and pregnant women are more likely to get sick with malaria** | | | |
| Strongly agree | 66.89 | 200 |  |
| Somewhat agree | 22.41 | 67 |  |
| Somewhat disagree | 3.01 | 9 |  |
| Strongly disagree | 3.68 | 11 |  |
| Don't Know | 4.01 | 12 |  |
| **You don't worry about malaria because it can be easily treated** | | |  |
| Strongly agree | 33.78 | 101 |  |
| Somewhat agree | 14.38 | 43 |  |
| Somewhat disagree | 21.07 | 63 |  |
| Strongly disagree | 29.77 | 89 |  |
| Don't Know | 1.00 | 3 |  |
| **Every case of malaria can potentially lead to death** | | |  |
| Strongly agree | 62.21 | 186 |  |
| Somewhat agree | 17.06 | 51 |  |
| Somewhat disagree | 9.70 | 29 |  |
| Strongly disagree | 10.37 | 31 |  |
| Don't Know | 0.67 | 2 |  |
| **Malaria in young children is very serious** |  |  |  |
| Strongly agree | 83.61 | 250 |  |
| Somewhat agree | 11.04 | 33 |  |
| Somewhat disagree | 3.34 | 10 |  |
| Strongly disagree | 0.67 | 2 |  |
| Don't Know | 1.34 | 4 |  |
| **Most people in this community never worry about getting malaria.** | | | |
| Strongly agree | 52.84 | 158 |  |
| Somewhat agree | 17.39 | 52 |  |
| Somewhat disagree | 8.70 | 26 |  |
| Strongly disagree | 14.38 | 43 |  |
| Don't Know | 6.69 | 20 |  |
| **Most people in this community only worry about getting malaria during the rainy season** | | | |
| Strongly agree | 60.87 | 182 |  |
| Somewhat agree | 17.73 | 53 |  |
| Somewhat disagree | 8.03 | 24 |  |
| Strongly disagree | 7.36 | 22 |  |
| Don't Know | 6.02 | 18 |  |
| **Most people in this community take their children to a health provider on the same day or the next day after he/she develops a fever.** | | | |
| Strongly agree | 61.87 | 185 |  |
| Somewhat agree | 18.73 | 56 |  |
| Somewhat disagree | 6.69 | 20 |  |
| Strongly disagree | 5.69 | 17 |  |
| Don't Know | 7.02 | 21 |  |
| **Most people in this community talk about malaria with their families** | | | |
| Strongly agree | 40.47 | 121 |  |
| Somewhat agree | 22.07 | 66 |  |
| Somewhat disagree | 13.04 | 39 |  |
| Strongly disagree | 11.37 | 34 |  |
| Don't Know | 13.04 | 39 |  |
| Total | 100.00 | 299 |  |
| **It is easy to tell whether a fever is malaria or not** | | |  |
| Strongly agree | 52.17 | 156 |  |
| Somewhat agree | 17.39 | 52 |  |
| Somewhat disagree | 9.70 | 29 |  |
| Strongly disagree | 18.06 | 54 |  |
| Don't Know | 2.68 | 8 |  |
| Total | 100.00 | 299 |  |
| **A person should take a child to a health provider the same day the child has a fever** | | | |
| Strongly agree | 79.93 | 239 |  |
| Somewhat agree | 16.05 | 48 |  |
| Somewhat disagree | 2.34 | 7 |  |
| Strongly disagree | 0.33 | 1 |  |
| Don't Know | 1.34 | 4 |  |
| Total | 100.00 | 299 |  |
| **When my child has a fever, I first go to a market to buy medicine for him/her** | | | |
| Strongly agree | 23.41 | 70 |  |
| Somewhat agree | 11.04 | 33 |  |
| Somewhat disagree | 17.06 | 51 |  |
| Strongly disagree | 47.83 | 143 |  |
| Don't Know | 0.67 | 2 |  |
| Total | 100.00 | 299 |  |
| **When my child has a fever, I first go to a pharmacy or chemist to buy medicine** | | | |
| Strongly agree | 24.75 | 74 |  |
| Somewhat agree | 11.71 | 35 |  |
| Somewhat disagree | 19.06 | 57 |  |
| Strongly disagree | 42.14 | 126 |  |
| Don't Know | 2.34 | 7 |  |
| Total | 100.00 | 299 |  |
| **The health provider is always the best person to talk to when you think your child has malaria** | | | |
| Strongly agree | 84.28 | 252 |  |
| Somewhat agree | 7.36 | 22 |  |
| Somewhat disagree | 6.02 | 18 |  |
| Strongly disagree | 1.67 | 5 |  |
| Don't Know | 0.67 | 2 |  |
| Total | 100.00 | 299 |  |
| **A person should consult with a “prophet” or religious leader to treat a child with fever.** | | | |
| Strongly agree | 20.74 | 62 |  |
| Somewhat agree | 13.38 | 40 |  |
| Somewhat disagree | 11.71 | 35 |  |
| Strongly disagree | 54.18 | 162 |  |
| Total | 100.00 | 299 |  |
| **It takes too much time to go see a health provider if a child has a fever** | | | |
| Strongly agree | 44.15 | 132 |  |
| Somewhat agree | 15.72 | 47 |  |
| Somewhat disagree | 13.71 | 41 |  |
| Strongly disagree | 25.08 | 75 |  |
| Don't Know | 1.34 | 4 |  |
| Total | 100.00 | 299 |  |
| **A person sick with fever is supposed to receive a blood test to confirm that the sickness is malaria before taking malaria drugs** | | | |
| Strongly agree | 84.28 | 252 |  |
| Somewhat agree | 11.37 | 34 |  |
| Somewhat disagree | 1.34 | 4 |  |
| Strongly disagree | 1.67 | 5 |  |
| Don't Know | 1.34 | 4 |  |
| Total | 100.00 | 299 |  |
| **A blood test for malaria is the only way to know if someone really has malaria** | | | |
| Strongly agree | 78.93 | 236 |  |
| Somewhat agree | 13.04 | 39 |  |
| Somewhat disagree | 5.35 | 16 |  |
| Strongly disagree | 2.01 | 6 |  |
| Don't Know | 0.67 | 2 |  |
| Total | 100.00 | 299 |  |
| **A health provider can be trusted when he/she says that a fever is not due to malaria** | | | |
| Strongly agree | 77.93 | 233 |  |
| Somewhat agree | 12.37 | 37 |  |
| Somewhat disagree | 6.69 | 20 |  |
| Strongly disagree | 3.01 | 9 |  |
| Total | 100.00 | 299 |  |
| **A health provider can be trusted when he/she says that a fever is due to ma** | | | |
| Strongly agree | 76.25 | 228 |  |
| Somewhat agree | 14.38 | 43 |  |
| Somewhat disagree | 5.35 | 16 |  |
| Strongly disagree | 3.01 | 9 |  |
| Don't Know | 1.00 | 3 |  |
| Total | 100.00 | 299 |  |
| **A person should only take malaria medicine if a health provider says that a fever is due to malaria** | | | |
| Strongly agree | 77.59 | 232 |  |
| Somewhat agree | 14.72 | 44 |  |
| Somewhat disagree | 4.01 | 12 |  |
| Strongly disagree | 3.68 | 11 |  |
| Total | 100.00 | 299 |  |
| **Modern medicine works better than traditional medicine** | | |  |
| Strongly agree | 71.24 | 213 |  |
| Somewhat agree | 14.38 | 43 |  |
| Somewhat disagree | 8.70 | 26 |  |
| Strongly disagree | 5.35 | 16 |  |
| Don't Know | 0.33 | 1 |  |
| Total | 100.00 | 299 |  |
| **It is important to take the entire course of malaria medicine to make sure the disease will be fully cured** | | | |
| Strongly agree | 84.95 | 254 |  |
| Somewhat agree | 9.70 | 29 |  |
| Somewhat disagree | 3.34 | 10 |  |
| Strongly disagree | 2.01 | 6 |  |
| Total | 100.00 | 299 |  |
| **When there is not enough money, it is more important that male children with fever get medicine rather than female children.** | | | |
| Strongly agree | 24.75 | 74 |  |
| Somewhat agree | 13.04 | 39 |  |
| Somewhat disagree | 11.04 | 33 |  |
| Strongly disagree | 49.83 | 149 |  |
| Don't Know | 1.34 | 4 |  |
| Total | 100.00 | 299 |  |
| **A person should still take malaria medicine even if the malaria test result** | | | |
| Strongly agree | 39.46 | 118 |  |
| Somewhat agree | 17.06 | 51 |  |
| Somewhat disagree | 12.71 | 38 |  |
| Strongly disagree | 28.76 | 86 |  |
| Don't Know | 2.01 | 6 |  |
| Total | 100.00 | 299 |  |
| **The malaria drugs obtained from the health facility are effective in treating malaria** | | | |
| Strongly agree | 75.59 | 226 |  |
| Somewhat agree | 15.72 | 47 |  |
| Somewhat disagree | 6.35 | 19 |  |
| Strongly disagree | 0.67 | 2 |  |
| Don't Know | 1.67 | 5 |  |
| Total | 100.00 | 299 |  |
| **The malaria medicine that you buy in the market is as good as the one distributed at the clinic** | | | |
| Strongly agree | 39.46 | 118 |  |
| Somewhat agree | 16.39 | 49 |  |
| Somewhat disagree | 9.70 | 29 |  |
| Strongly disagree | 29.10 | 87 |  |
| Don't Know | 5.35 | 16 |  |
| Total | 100.00 | 299 |  |
| **ACTs work quickly to treat malaria** |  |  |  |
| Strongly agree | 54.18 | 162 |  |
| Somewhat agree | 16.39 | 49 |  |
| Somewhat disagree | 3.68 | 11 |  |
| Strongly disagree | 5.02 | 15 |  |
| Don't Know | 20.74 | 62 |  |
| Total | 100.00 | 299 |  |
| **When the entire course of malaria medicine is taken, the disease will be cured** | | | |
| Strongly agree | 77.93 | 233 |  |
| Somewhat agree | 12.71 | 38 |  |
| Somewhat disagree | 5.35 | 16 |  |
| Strongly disagree | 2.34 | 7 |  |
| Don't Know | 1.67 | 5 |  |
| Total | 100.00 | 299 |  |
| **Generally, how many people in your community take their children to a health provider on the same day or day after they develop a fever?** | | | |
| All people | 18.73 | 56 |  |
| Most people | 41.47 | 124 |  |
| More than half | 14.72 | 44 |  |
| Fewer than half | 11.71 | 35 |  |
| Don't Know | 13.38 | 40 |  |
| Total | 100.00 | 299 |  |
| **Generally, how many children in your community are taken to a health facility with fever get tested for malaria?** | | | |
| All people | 21.07 | 63 |  |
| Most people | 41.14 | 123 |  |
| More than half | 14.72 | 44 |  |
| Fewer than half | 10.03 | 30 |  |
| Don't Know | 13.04 | 39 |  |
| Total | 100.00 | 299 |  |
| **In your community, how frequently do health facilities have the tests for malaria?** | | | |
| Always | 24.75 | 74 |  |
| Most of the time | 36.45 | 109 |  |
| Sometimes | 17.73 | 53 |  |
| Rarely | 8.36 | 25 |  |
| Don't Know | 12.71 | 38 |  |
| Total | 100.00 | 299 |  |
| **In your community, how frequently do health facilities have the medicines for treating malaria?** | | | |
| Always | 19.06 | 57 |  |
| Most of the time | 41.81 | 125 |  |
| Sometimes | 16.05 | 48 |  |
| Rarely | 9.70 | 29 |  |
| Don't Know | 13.38 | 40 |  |
| Total | 100.00 | 299 |  |
